# Induction of chronic destructive arthritis in SCID mice by arthritogenic fibroblast-like synoviocytes derived from mice with antigen-induced arthritis

**DOI:** 10.1186/s13075-018-1720-y

**Published:** 2018-11-22

**Authors:** Oliver Frey, Marion Hückel, Mieczyslaw Gajda, Peter K. Petrow, Rolf Bräuer

**Affiliations:** 1 Institute of Pathology, University Hospital, Jena, Germany; 2 Institute of Clinical Chemistry and Laboratory Medicine, University Hospital, Am Klinikum 1, D-07743 Jena, Germany; 3 Present address: Institute of Medical Diagnostics, Berlin, Germany

**Keywords:** Arthritis, Synovial fibroblast, Joint destruction

## Abstract

**Background:**

Fibroblast-like synoviocytes (FLSs) from patients with rheumatoid arthritis (RA) are autonomously activated to maintain inflammation and joint destruction in co-transplantation models. To elucidate inducing mechanisms involved in this altered behavior, the arthritogenic potential of FLSs from murine antigen-induced arthritis (AIA) were investigated in a transfer model.

**Methods:**

FLSs were isolated, expanded in vitro, and transferred into knee joint cavities of severe combined immunodeficient (SCID) mice. Their arthritogenic capacity was assessed by monitoring joint swelling and evaluation of histological parameters 70 to 100 days after transfer.

**Results:**

FLSs from AIA mice were able to transfer arthritis into recipient SCID mice. FLS transfer induced a chronic arthritis with recruitment of inflammatory cells and marked cartilage destruction. Long-lasting inflammation was not required for imprinting of arthritogenicity in FLSs since cells isolated from acute arthritic joints were fully competent to transfer arthritis. We also observed arthritogenic potential in FLSs isolated from contralateral non-arthritic joints in our monoarticular arthritis model.

**Conclusions:**

We show that the transformation of FLSs into arthritogenic cells occurs early in arthritis development. This challenges current hypotheses on the role of these cells in arthritis pathogenesis and opens up the way for further mechanistic studies.

## Background

Rheumatoid arthritis (RA) is a chronic inflammatory disease that primarily affects the joints. Key histological features of RA are infiltration by cells from the innate and the adaptive immune system, hyperplasia of the synovial membrane, and destruction of cartilage and adjacent bone [1, 2]. Fibroblast-like synoviocytes (FLSs) are the dominant cell type in the hyperplastic synovial membrane and are thought to play a key role in the pathogenesis of RA. Via their production of different inflammatory cytokines and chemokines, they can recruit inflammatory cells and sustain their persistence in inflamed joints. FLSs also produce matrix-degrading enzymes such as matrix metalloproteases (MMPs) and cathepsins and are thereby directly involved in joint destruction. A remarkable feature of FLSs is their ability to maintain their activated phenotype in tissue culture or upon engraftment in severe combined immunodeficient (SCID) mice [3–5]. Early models used transplantation of whole RA synovial tissue to study the inflammatory and destructive features of synovial cells in an in vivo environment [6–8]. Since synovial membranes of RA patients contain a mixture of different cell types, in later studies FLSs were isolated prior to transfer into SCID mice [9, 10]. In such chimeric models, FLSs attach to and invade the co-implanted cartilage, and they recruit inflammatory cells. Furthermore, destructive fibroblasts migrate to and destroy unaffected cartilage co-transplanted in other anatomical locations upon transfer in immunodeficient mice [11]. Thus, although this model does not reflect all aspects of the complex pathogenesis of RA, it fosters the view that a permanent and autonomous activation of FLSs is central to disease progression.

Since the immunological process which finally triggers RA can precede the clinical disease onset by many years, the cascade of events eventually leading to FLS activation and disease cannot be reconstructed from patient material. Thus, animal models are clearly needed for a better understanding of the contribution of fibroblasts to chronic inflammation and joint destruction in arthritis. So far, there is only one published study using cloned immortalized FLSs confirming the observation of an arthritogenic behavior of FLSs in a tumor necrosis factor (TNF)-transgenic mouse model [5]. It is unknown whether this behavior can also be imprinted to non-immortalized FLSs isolated from arthritic wild-type mice.

Based on the above-mentioned studies using FLSs from patients with RA, it is widely assumed that the arthritis-promoting effect of these cells is the consequence of long-standing chronic inflammation. This has not yet been proven experimentally, and we therefore aimed to elucidate if chronic inflammation is mandatory for arthritogenic transformation of FLSs in our mouse model. If an acute (short-term) arthritis were sufficient to induce persistent changes in FLS behavior, it could be hypothesized that these cells are rather accomplices than villains in the induction and perpetuation of chronic arthritis. Thus, our study aimed at answering some fundamental questions of the biology of FLSs for the pathogenesis of arthritis.

We used antigen-induced arthritis (AIA), which is induced by intraarticular injection of an antigen into the knee joint cavity of animals preimmunized with the same antigen. This model was chosen because of its 100% incidence and the well-documented time course of pathogenic changes upon arthritis triggering [12, 13]. These features allows for a systematic study of FLS behavior isolated from arthritic as well as non-arthritic knee joints.

## Methods

### Animals and arthritis induction

Female C57BL/6 mice aged 6–12 weeks were immunized 21 and 14 days before arthritis induction by subcutaneous injection of 100 μg methylated bovine serum albumin (mBSA, Sigma, Deisenhofen, Germany) in 50 μl saline, emulsified in 50 μl complete Freund’s adjuvant (CFA; Sigma), supplemented with 2.0 mg/ml heat-killed *Mycobacterium tuberculosis* (strain H37RA, Becton Dickinson, Heidelberg, Germany). At the same time, mice were injected with 5 × 10^8^ heat-killed *Bordetella pertussis* bacteria (Chiron Behring, Marburg, Germany). On day 0, arthritis was induced by a single injection of 100 μg mBSA in 25 μl saline into the right knee joint cavity. The left knee joint was left untreated. The animals were sacrificed, and the knees were dissected for the preparation of synovial cells in acute (before day 7) or chronic phase (after day 7) of AIA.

BALB/c SCID mice (Harlan Winkelmann, Borchen, Germany) aged 6–8 weeks were used as recipients. The SCID mice were housed in isolated cages at the Central Animal Facility, University Hospital Jena.

All experiments were approved by the appropriate governmental authority (Thüringer Landesamt für Verbraucherschutz) and conducted in accordance with institutional and state guidelines.

### Preparation of FLSs

FLSs were obtained by explant cultures from synovial tissue dissected from the knee joints of normal, immunized, or arthritic mice (at different time points after arthritis induction) as previously described in [14]. The tissue was digested with 0.1% trypsin (Boehringer Mannheim, Germany) in phosphate-buffered saline (PBS) for 30 min and after washing, digested with 0.1% collagenase P (Boehringer) in PBS for an additional 2 h. The culture was maintained in Dulbecco’s modified Eagle’s medium (DMEM), completed with 10 mM 4-(2-hydroxyethyl)-1-piperazineethanesulfonic acid (HEPES), 1 mM sodium pyruvate (all from Gibco BRL, Gaithersburg, MD, USA), 100 U/ml penicillin (Jenapharm, Jena, Germany), 0.1 mg/ml streptomycin (Grünenthal, Stolberg, Germany), 2 mM glutamine (Gibco), and 20% fetal calf serum (FCS; Gibco) for 7 days at 5% CO_2_ and 37 °C. Medium exchange took place each day. Synovial cells emerged from explanted synovium within 7 days. Confluent FLSs were detached by digestion with 0.25% trypsin/0.02% ethylenediaminetetraacetic acid (EDTA; Gibco) and subcultured in complete DMEM supplemented with 10% FCS. Synovial cells were repeatedly passaged to enrich FLSs and to deplete macrophages [10, 15]. For some experiments we depleted macrophages from the cell cultures after the first passage with magnetic cell sorting using anti-CD11b (clone 5C6; Serotec, Oxford, UK) followed by anti-rat Dynabeads® (10 beads/cell; Dynal, Hamburg, Germany). The separation was achieved using a Magnetic Particle Concentrator (Dynal MPC®). All FLS preparations used for transfer experiments contained > 95% fibroblasts by their typical spindle-shaped morphology.

Cell cultures were routinely screened for infection with mycoplasma (*Mycoplasma orale, M. arginini, M. hyorhinis*, and *M. laidlawii)* with an enzyme-linked immunosorbent assay (ELISA) detection kit (Roche Diagnostics, Mannheim, Germany), according to the manufacturer’s instructions.

### Cell transfer and monitoring of arthritis development

FLSs of the third to the fifth passage were harvested subsequently using 0.25% trypsin/0.02% EDTA in PBS and washed and suspended in PBS at a final concentration of 1.2 × 10^7^ cell/ml; then 25 μl (3 × 10^5^ cells) of this suspension were injected into the knee joint cavity of recipient SCID mice.

Development of clinical arthritis in SCID mice was monitored by measuring the mediolateral knee joint diameter with an Oditest vernier caliper (Kroeplin Längenmesstechnik, Schlüchtern, Germany). Knee joint swelling was expressed as the difference between the right (injected) and left (untreated) knees.

For histological assessment of arthritis severity [14], SCID mice were sacrificed 70–100 days after cell transfer (time indicated in results). Total knee joints were removed and fixed in 4.5% Tris-buffered formalin. After decalcification with 15% EDTA, embedding in paraffin, and sectioning, slides were stained with hematoxylin and eosin (H&E). A minimum of three sections (2 μm) from three different levels of the knee joints were evaluated blindly by at least two different observers (MG, PKP) for the degree of inflammation and joint destruction. The severity of inflammation was evaluated on a 0–3 point scale indicating lining layer hyperplasia and cellular infiltration (0: none, 1: mild, 2: moderate, 3: strong changes). The severity of degradation was assessed by scoring pannus formation and chondrocyte necrosis as well as cartilage and bone erosion. A final arthritis score was calculated for each animal by adding the scores for all four parameters (upper limit grade 12).

For the evaluation of proteoglycan loss, sections were stained with Safranin O. In this method, the degree of staining is inversely correlated with the loss of proteoglycans in the cartilage layers.

### Statistics

All data shown are mean ± standard error of mean. Statistically significant differences were calculated using the non-parametric Mann-Whitney *U* test (two-tailed) with SPSS 22 (IBM). *p* values less than 0.05 were considered as statistically significant.

## Results

### Transfer of FLSs from C57BL/6 mice with AIA induce arthritis in SCID mice recipients

**Fig. 1:**
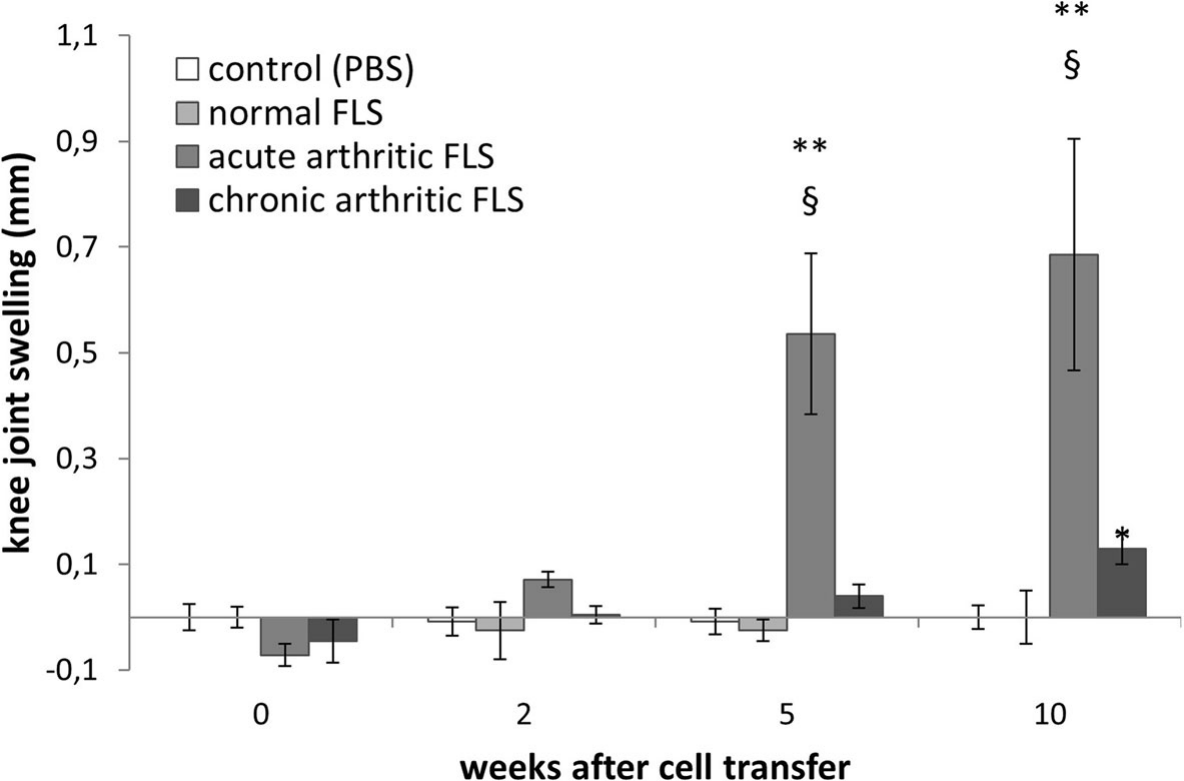
Knee joint swelling in SCID mice after transfer of fibroblast-like synoviocytes (FLSs). FLSs from healthy C57BL/6 mice (normal FLSs, *n* = 5), from C57BL/6 mice with acute AIA (7 days, acute arthritic FLSs, *n* = 7), or from those with chronic AIA (21 days, chronic arthritic FLSs, *n* = 11) were transferred into the right knee joint cavity of SCID mice. Buffer was injected as a control (control, PBS, *n* = 6). Joint swelling was evaluated by differences between joint diameter of right knee (ispilateral) and left knee (contralateral) at day of measurement. All data shown are mean ± standard error of mean. (* *p* < 0.05 vs. PBS, ** *p* < 0.01 vs. PBS, § *p* < 0.05 vs. normal FLSs; Mann-Whitney *U* test.) Data shown are a representative example of at least two independent experiments per group

In order to investigate the arthritogenic potential of FLSs in vivo, these cells were injected into the knee joints of SCID mice. We followed arthritis development by measurement of the knee joint diameter and histological examination of the knee joints at the end point of the experiment. As shown in Fig. 1, FLSs from affected knee joints of mice with acute arthritis (isolated until day 7 post AIA induction) induced a slowly growing increase in joint swelling in the recipients. This surrogate parameter of joint inflammation was also found in recipients of FLSs from mice with chronic AIA (isolated after day 7), albeit to a lesser extent. We did not detect a similar knee joint swelling in SCID mice injected with PBS or FLSs isolated from naïve, non-arthritic mice, suggesting that the arthritogenic capacity is restricted to FLSs isolated from arthritic mice.

**Fig. 2:**
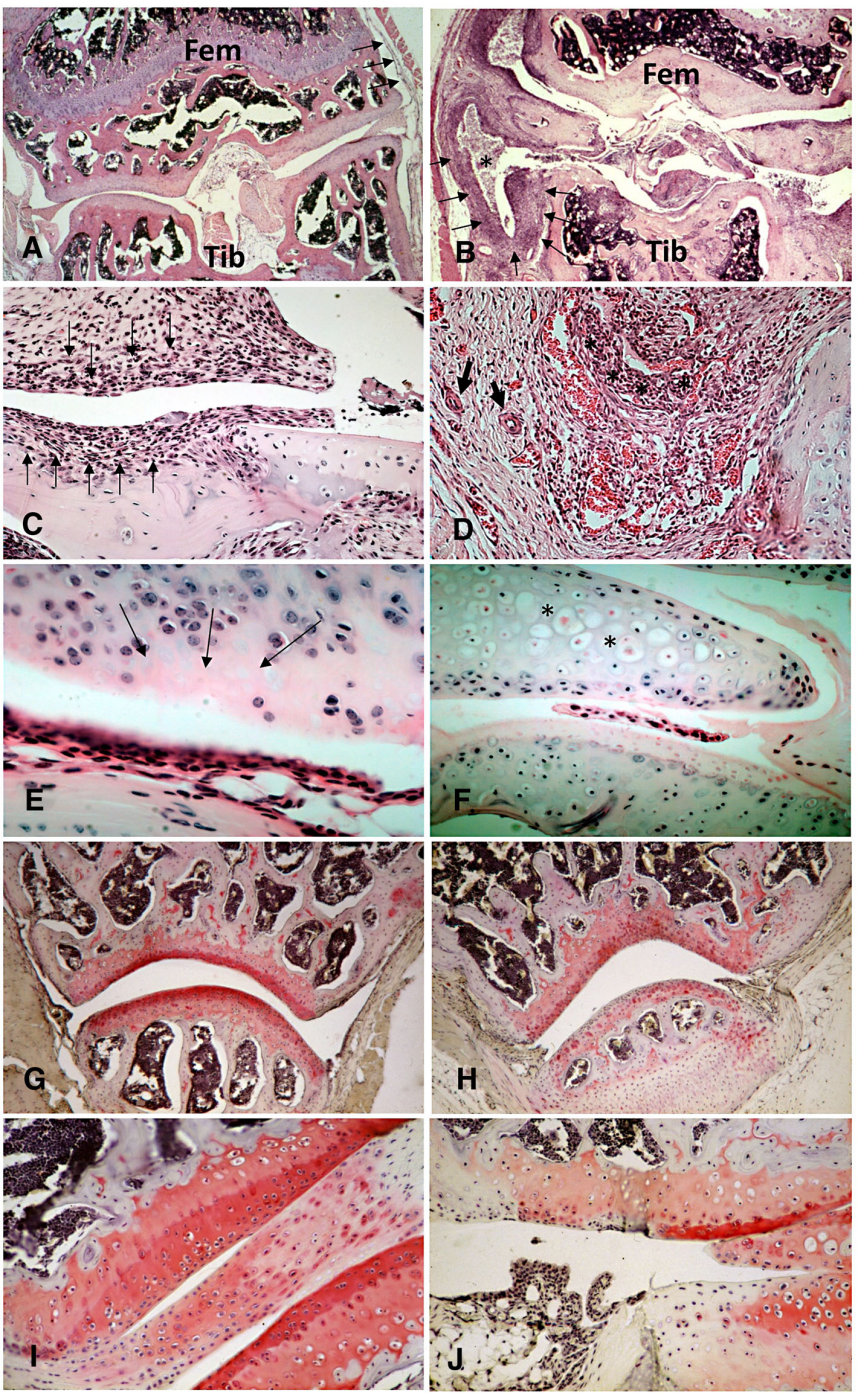
Histological examination of knee sections from SCID mice. **a** Sections from healthy untreated knee joint (*Fem* femur, *Tib* Tibia). *Arrows* indicate a normal synovial membrane. **b** Knee joints 100 days after transfer of FLSs isolated from C57BL/6 mice with acute AIA. Shown is an overview of the inflamed and destructed joint (*Fem* femur, *Tib* tibia). *Arrows* indicate the hyperplastic and inflamed synovial membrane. *Asterisks* indicate the inflammatory exudate in the knee cavity. **c** and **d** Higher magnification to illustrate hyperplasia of the synovial lining layer and pannus formation (*arrows*) and inflammatory infiltration (*asterisks*) and neovascularization (*thick arrows*). **e** and **f** Cartilage erosions (*arrows*) and necrosis of chondrocytes (*asterisks*). Staining of proteoglycans with Safranin O in healthy knee joint sections (**g**, **i**) and arthritic knee joint of SCID mice (**h**, **j**) 100 days after cell transfer shows loss of proteoglycans in arthritic SCID joints. Sections **a**–**f** were stained with H&E, **g**–**j** with Safranin O. Magnifications: **a**, **b**, **g**, **h** 40×, **i**, **j** 100×, **c**, **d**, **f** 200×, **e** 400×

**Fig. 3:**
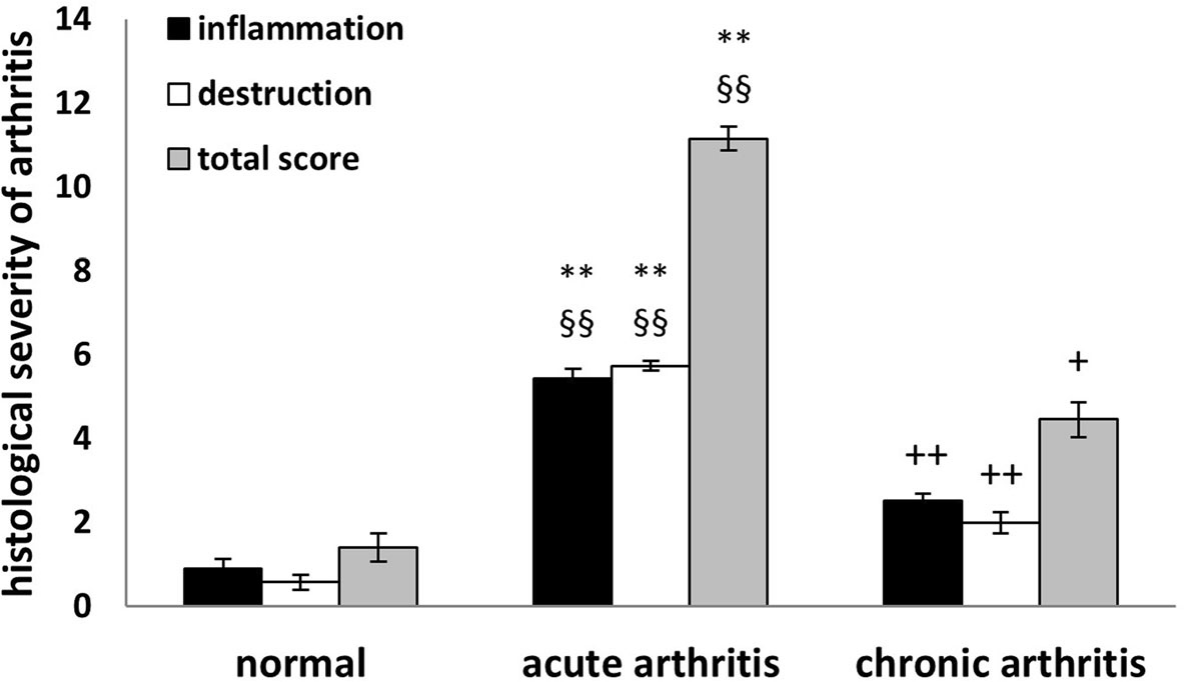
Semiquantitative histologic scoring of inflammation, joint destruction, and total arthritis score in knee joint sections from SCID mice 70–100 days after transfer of FLSs. FLSs were isolated from non-immunized, non-arthritic mice (normal, *n* = 9), mice with acute arthritis (up to day 7 post AIA induction, *n* = 13), or mice with chronic AIA (isolated after day 7 post AIA induction, *n* = 52). Data are pooled from at least two experiments. Histological scoring was performed by two independent investigators on a 0–3 point scale for synovial lining layer hyperplasia, cellular infiltration, pannus formation, and cartilage invasion, giving a total score of 12. All data shown are mean ± standard error of mean (** *p* < 0.001 acute vs. normal, §§ *p* < 0.001 acute vs. chronic, + *p* < 0.01, ++ *p* < 0.001 chronic vs. normal, Mann-Whitney *U* test)

Histological evaluation of joint sections confirmed arthritis development observed by joint swelling. As shown in Fig. 2, injection of FLSs isolated from C57BL/6 mice with acute arthritis into SCID recipients resulted in cellular infiltration, hyperplasia of the synovial lining layer, pannus formation, and chondrocyte necrosis as well as cartilage and bone erosions. Safranin O staining revealed that the injection of arthritogenic cells also caused loss of proteoglycans. These typical histological signs of arthritis were not seen in recipients of FLSs isolated from naïve, non-arthritic mice. Semiquantitative scoring of histological arthritis severity showed a higher degree of inflammation and joint destruction, resulting in a higher total arthritis score, in the recipients from FLSs isolated from mice with acute compared to chronic arthritis (Fig. 3). Recipients of FLSs from naïve, non-arthritic mice showed only very limited signs of inflammation, while joint erosions were almost completely absent.

Thus, our data indicate that FLSs isolated from mice with AIA possess and maintain their arthritogenic potential without exogenous activation, a behavior which is similar to that of FLSs isolated from patients with RA. Remarkably, this arthritogenic potential of FLSs is induced early in the disease course and is not a consequence of long-lasting inflammation.

### Depletion of macrophages prior to transfer had no influence on disease outcome

**Fig. 4:**
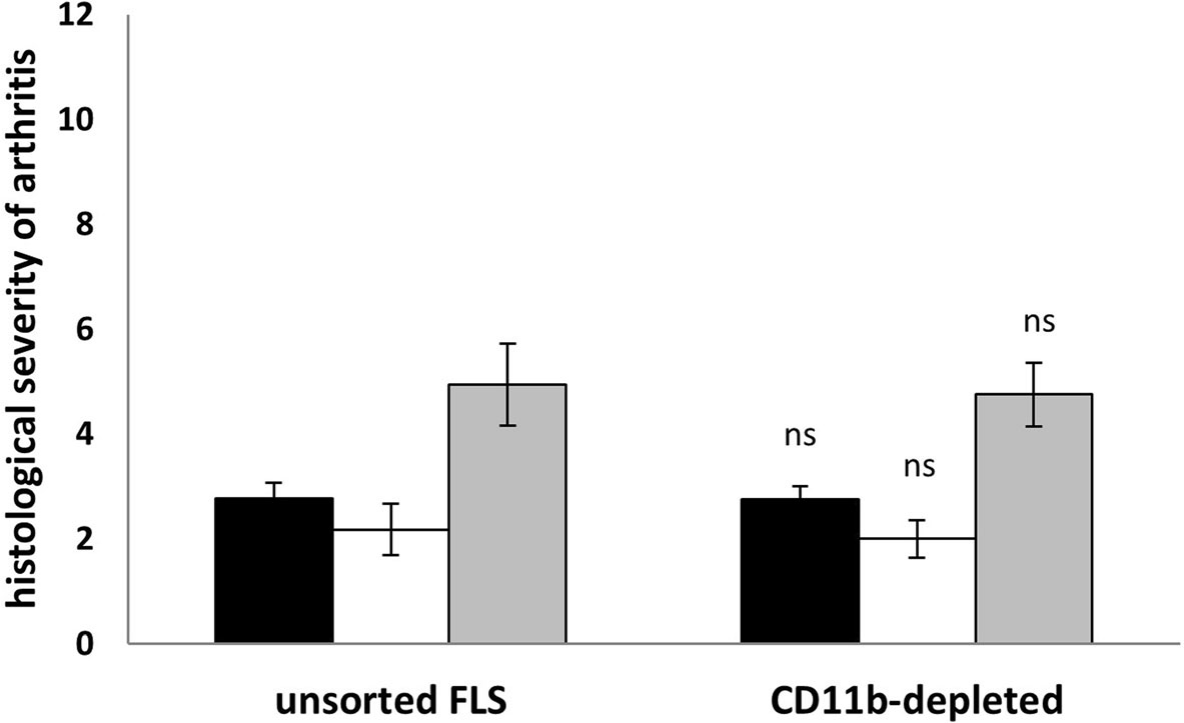
Histological arthritis severity in recipient SCID mice that were intraarticularly injected with total unsorted FLSs or with CD11b-depleted FLSs (isolated 5 or 8 days post AIA induction). Cultures of depleted and non-depleted FLSs as well as cell transfer were done in parallel. Black bars: inflammation, white bars: joint destruction, grey bars: total arthritis score. All data shown are mean ± standard error of mean (total cells *n* = 17, depleted *n* = 28, *ns* no significant differences). Data are pooled from two independent experiments

Our FLS cultures still contained approximately 20% CD11b^+^ macrophage-like cells after the third passage (data not shown). It is possible that these activated macrophages and not the FLSs contained in these preparations could trigger inflammation and joint destruction in the recipient SCID mice knee joints. We therefore depleted CD11b^+^ cells using immunobeads from FLS preparations from acutely arthritic AIA mice. Arthritogenicity of these pure FLS preparations (< 1% CD11b^+^, data not shown) were compared with unsorted FLS preparations in transfer experiments. The experiments were performed identically to those previously described. As shown in Fig. 4, we observed no differences in inflammatory changes or joint destruction upon histological examination of the recipient knee joints. These data show that the presence of macrophages has no effect on the arthritogenicity of FLS preparations from acutely arthritic joints from AIA mice. Furthermore, the small enrichment of fibroblasts in macrophage-depleted preparations did not lead to a significant increase of arthritis severity.

### FLSs from contralateral (control) knee of AIA mice also show arthritogenic potential

**Fig. 5:**
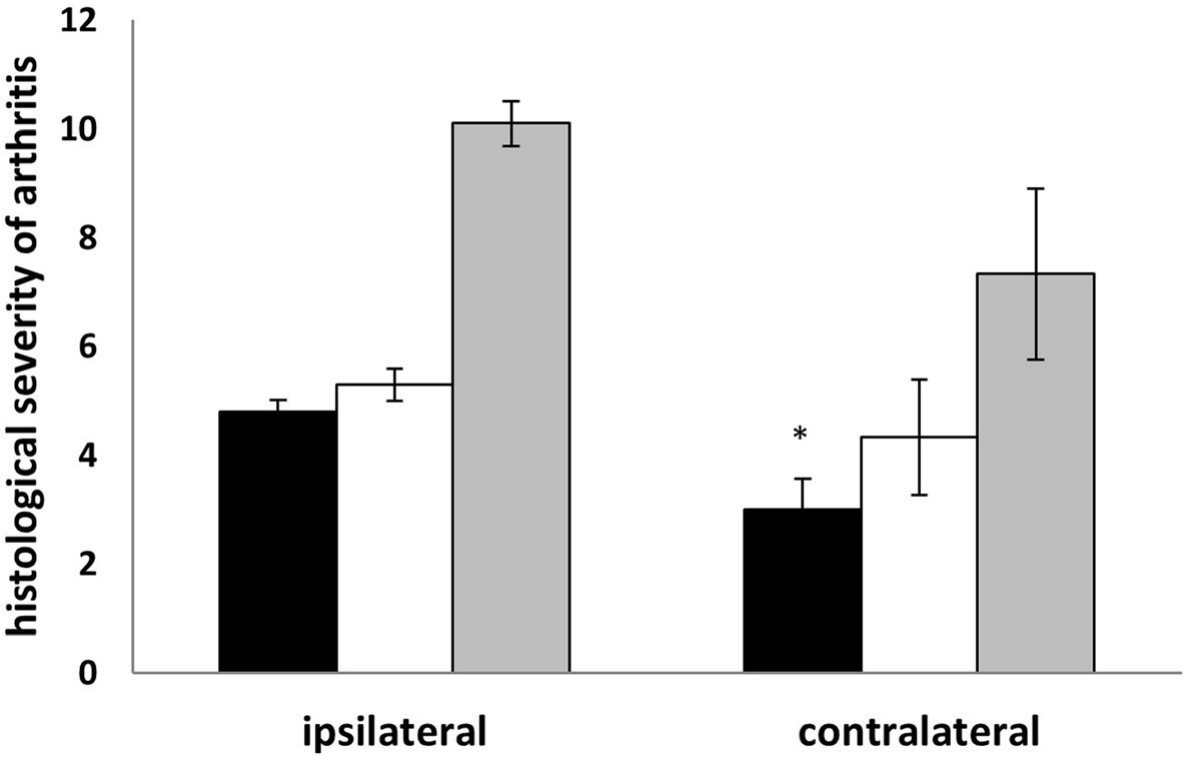
Histological arthritis scoring after transfer of FLSs from the arthritic and contralateral knee joints of AIA mice. Cells were isolated and transferred in parallel, and the data shown are pooled from three independent experiments using FLSs from different time points (day 8 or 28) after AIA induction. Black bars: inflammation, white bars: joint destruction, grey bars: total arthritis score. All data shown are mean ± standard error of mean (ipsilateral *n* = 10, contralateral *n* = 6 (* *p* < 0.05 ipsilateral vs. contralateral)

Having demonstrated that the imprinting of arthritogenic behavior on FLSs occurs early during arthritis development, we next examined if interactions between fibroblasts and infiltrating cells are necessary for the induction of FLS aggressiveness. Since AIA is a monoarticular arthritis model, we are able to compare the arthritogenicity of FLS preparations from affected ipsilateral joints with the non-affected contralateral knee joints. Although the contralateral knee joint in AIA never shows histological abnormalities, FLS preparations from these joints were surprisingly potent inducers of arthritis. Semiquantitative scoring of end-point histology of the recipient knee joints showed significantly reduced inflammation but similar joint destruction in recipients of FLSs from ipsilateral and contralateral knee joints (Fig. 5).

## Discussion

In this paper we show that FLSs isolated from knee joints of mice with AIA have the capacity to cause severe inflammation and destruction when injected intraarticularly into knee joints of SCID mice. Isolated FLSs express their arthritogenic potential even after several passages in vitro, indicating a long-lasting transformation to autonomous aggressor cells. This arthritogenicity was intrinsic to FLSs, since removal of contaminating macrophages did not affect arthritis severity in the recipients.

These features resemble the aggressive behavior of human FLSs from RA patients which upon co-transfer with engrafted cartilage into SCIID mice possess pro-inflammatory and pro-destructive activity [3, 9, 16]. In vitro studies have demonstrated a loss of contact inhibition as well as constitutive expression of cytokines and matrix-degrading enzymes as causative for the arthritogenicity of RA FLSs (for a review see [17]). However, the mechanisms leading to their transformation from cells with mainly homeostatic functions into cells with such an altered phenotype are still a matter of debate. Somatic mutations in genes regulating cellular functions like proliferation and apoptosis have been implicated in cell-autonomous aggressive behavior. Also, epigenetic alterations have been identified in RA FLSs, which may be imprinted by chronic exposure to inflammatory cytokines or cells [17]. Our findings presented here show that FLSs isolated from mice in the acute stage of AIA were already able to induce inflammation and joint destruction in the recipients. Compared to FLSs isolated from mice with chronic AIA they even induced a more severe arthritis in the recipients. This higher arthritogenicity of acute-stage FLSs might be related to their higher level of activation. Previous work from our group has demonstrated higher expression of matrix-degrading enzymes and concomitant maximal proteoglycan depletion in the acute stage of arthritis [18]. Taken together this indicates that the activation and transformation of FLSs is an early event in the disease course in AIA. An early upregulation of MMP-3 and MMP-9 activities has been described in TNF-overexpressing mice that develop arthritis [19], also implying that FLS activation is an early event in arthritis pathogenesis and supporting our findings.

Although the activation and transformation of FLSs in RA might be multifactorial, our data imply that somatic mutations might not be an ultimate prerequisite for their arthritogenicity, but instead short-term activation through inflammatory mediators might be sufficient [3, 20–25].

Moreover, we have demonstrated that FLSs isolated from the contralateral, non-affected joints in AIA can induce inflammation and joint destruction in our transfer system. This intriguing and unexpected finding implies that activation and transformation of FLSs can occur in the absence of infiltrating inflammatory cells. Although AIA is considered to be a monarthritis, several pieces of evidence suggest at least a partial affection of the contralateral joints. In previous work we have demonstrated a depletion of cartilage proteoglycans in non-affected joints in AIA in rats [26, 27]. We also detected macrophage activation by immunohistochemistry and near-infrared molecular imaging in the contralateral joint of arthritic mice [28], presumably as a consequence of the systemic macrophage activation in AIA [29]. Supernatants of synoviocyte cultures from contralateral joints of rats with AIA contained higher levels of interleukin (IL)-6 than those of healthy controls [30]. Although the exact reasons for activation of the contralateral FLSs in AIA remain elusive, this could be explained by elevated systemic levels of cytokines like TNF or IL-6 induced by immunization and intraarticular antigen challenge. Overexpression of TNF or enhanced IL-6 signaling due to a gain-of-function mutation of gp130 is sufficient to induce arthritis in mice [31, 32]. Strikingly, both cytokines can still induce joint inflammation when the expression of their receptor is restricted to mesenchymal cells such as FLSs [19, 32]. These data clearly demonstrate that activation of FLSs via cytokines is solely sufficient to induce arthritis.

Other activation stimuli could be delivered via neuronal mechanisms. Receptors for neurokinin 1 and bradykinin 2 are bilaterally upregulated in dorsal root ganglia of rats with AIA [27]. It has been shown that neurokinin 1 can transmit signals responsible for cartilage destruction at distal sites after induction of a unilateral local inflammation [33]. Unilateral arthritis also leads to bilateral hyperalgesia that in turn could trigger inflammation on the contralateral site via spinal reflexes [34]. Furthermore, the enhanced hippocampal neurogenesis found in AIA is mainly driven by immunization, not by localized inflammation [35]. Shenker et al. proposed that these contralateral effects in many models are mediated through neural mechanisms rather than reflecting a systemic or circulatory effect [36].

Regardless of whether the activation of FLSs is mediated via systemic or neuronal mechanisms, the fact that such a remote activation can occur is intriguing and could have implications for our understanding of RA pathogenesis. Assuming that such a remote activation of FLSs also occurs in humans, it is possible that FLS transformation is an early event in arthritis pathogenesis rather than a consequence of long-lasting local inflammation. In other words, FLS activation by such stimuli could be the missing link between systemic alterations of the immune system and joint-specific inflammation. Multiple animal models in which arthritis is induced by autoimmunity against ubiquitous antigens [37–40], by defective apoptosis [41], by a generally more autoreactive T cell repertoire [42], or by increased cytokine signaling [31, 43–45] provide evidence that the recognition of joint-specific antigens is not an absolute requirement for joint-specific inflammation (for a review see [46]). Taking into account that biomarkers of inflammation are upregulated years before clinical disease onset, such mechanisms could also be a pathogenic principle in human RA [47, 48].

While the FLSs from contralateral joints are nearly as equally potent inducers of inflammation and joint destruction as FLSs from arthritic AIA joints in our transfer system, there is little or no inflammation and only limited joint destruction in the joints from which the cells originate. This could be explained by an active counter-regulation in situ in the joints of the donor mice, which is intriguing, because the identification of such a regulatory mechanism could open up the way for new therapeutic approaches.

## Conclusions

In summary, we have demonstrated in this report that FLSs from mice with experimental inflammatory AIA share important features with FLSs from patients with human RA. Therefore, this model is a valuable tool for experimental analyses of the molecular mechanisms of the pathogenic role of FLSs in the induction and joint destruction in chronic arthritis.

## Abbreviations

AIA: Antigen-induced arthritis
FLS: Fibroblast-like synoviocyte
MMP: Matrix metalloprotease
RA: Rheumatoid arthritis
SCID: Severe combined immunodeficient
TNF: Tumor necrosis factor
